# *Dnmt3b* Deficiency in Myf5^+^-Brown Fat Precursor Cells Promotes Obesity in Female Mice

**DOI:** 10.3390/biom11081087

**Published:** 2021-07-23

**Authors:** Shirong Wang, Qiang Cao, Xin Cui, Jia Jing, Fenfen Li, Huidong Shi, Bingzhong Xue, Hang Shi

**Affiliations:** 1 Department of Biology, Georgia State University, Atlanta, GA 30303, USA; swang38@student.gsu.edu (S.W.); caoqiang78@gmail.com (Q.C.); xcui@gsu.edu (X.C.); jjing@gsu.edu (J.J.); fli3@gsu.edu (F.L.); 2 Georgia Cancer Center, Medical College of Georgia, Augusta University, Augusta, GA 30912, USA; HSHI@augusta.edu

**Keywords:** Dnmt3b, epigenetics, brown adipocytes, thermogenesis, obesity

## Abstract

Increasing energy expenditure through activation of brown fat thermogenesis is a promising therapeutic strategy for the treatment of obesity. Epigenetic regulation has emerged as a key player in regulating brown fat development and thermogenic program. Here, we aimed to study the role of DNA methyltransferase 3b (Dnmt3b), a DNA methyltransferase involved in de novo DNA methylation, in the regulation of brown fat function and energy homeostasis. We generated a genetic model with *Dnmt3b* deletion in brown fat-skeletal lineage precursor cells (3bKO mice) by crossing *Dnmt3b*-floxed (fl/fl) mice with *Myf5-Cre* mice. Female 3bKO mice are prone to diet-induced obesity, which is associated with decreased energy expenditure. Dnmt3b deficiency also impairs cold-induced thermogenic program in brown fat. Surprisingly, further RNA-seq analysis reveals a profound up-regulation of myogenic markers in brown fat of 3bKO mice, suggesting a myocyte-like remodeling in brown fat. Further motif enrichment and pyrosequencing analysis suggests myocyte enhancer factor 2C (*Mef2c*) as a mediator for the myogenic alteration in *Dnmt3b*-deficient brown fat, as indicated by decreased methylation at its promoter. Our data demonstrate that brown fat *Dnmt3b* is a key regulator of brown fat development, energy metabolism and obesity in female mice.

## 1. Introduction

Obesity poses a serious health threat to the current society due to its ability to increase mortality and morbidities including various metabolic disorders, such as type 2 diabetes, hypertension, dyslipidemia, and cardiovascular diseases [1]. A net energy influx resulting from energy intake over expenditure leads to obesity [1]. Brown fat is a key contributor to whole body energy expenditure due to its ability to generate adaptive thermogenesis [2,3]. Brown fat is capable of producing heat due to its unique expression of UCP1, a mitochondrial inner membrane protein that uncouples oxidative phosphorylation from ATP synthesis, thereby profoundly increasing energy expenditure [1,4,5,6]. Emerging evidence also supports the existence of UCP1-independent thermogenesis mediated by the sarco/endoplasmic reticulum Ca^2+^-ATPase 2b/ATPase, Ca^2+^ transporting, cardiac muscle, slow twitch 2 (SERCA2b/ATP2a2)-mediated calcium cycling or the creatine-driven substrate cycling [7,8]. Rodents possess two distinct types of thermogenic adipocytes: the classic brown adipose tissue (BAT) that mainly resides in the confined areas such as interscapular region (iBAT) and inducible beige adipocytes that are dispersed in white adipose tissue (WAT) and can be induced by β-adrenergic activation in response to cold challenge or β adrenergic agonists [2,9,10,11,12,13]. Brown and beige adipocyte thermogenesis has been shown as a promising therapeutic target for the treatment of obesity [14,15,16]. The recent discovery of thermogenic brown fat in humans implies that induction of brown and beige fat thermogenesis is a promising therapeutic strategy to combat obesity [17,18,19].

Obesity is a polygenic disease that results from the interaction between genes and environmental factors (e.g., diets). Environmental factors often affect the expression of genes involved in metabolism through reprogramming epigenomes. A growing body of evidence has suggested that Epigenetic regulation plays a key role in the regulation of metabolic pathways underlying the development of obesity and its associated disorders [20,21]. DNA methylation is a common epigenetic regulation that involves a transfer of a methyl group to cytosine typically at CpG sites. DNA methylation is commonly seen at the genes’ promoter and 5′ region, where CpGs are often clustered to form islands [22,23]. DNA hypermethylation at the gene promoter tends to cause gene silencing, whereas DNA hypomethylation is associated with activated gene expression [23]. Three functional DNA methyltransferases (DNMTs), including DNMT1, 3a and 3b, have been identified [23]. DNMT1 preferentially catalyzes the methylation of hemimethylated DNA to maintain the methylation pattern during DNA replication, whereas Dnmt3a and 3b are often involved in de novo methylation using unmethylated DNA as a substrate [23]. We recently discovered several epigenetic pathways, including DNA methylation, as an important regulator of white adipocyte differentiation and brown adipocyte thermogenic program [24,25,26,27]. For instance, we reported that DNMT1 and 3a both play a biphasic role in regulating 3T3-L1 adipogenesis [24]. In addition, we also discovered that brown fat Dnmt1 or 3a is important in cold-induced thermogenesis and diet-induced obesity in mice [28]. In this study, we aimed to study the role of Dnmt3b in regulating brown fat thermogenic function and energy metabolism. We generated a genetic model with *Dnmt3b* knockout in brown fat-skeletal lineage precursor cells (3bKO) by crossing *Dnmt3b*-floxed mice (fl/fl) with myogenic factor 5 (*Myf5*)*-Cre* mice. We then characterized metabolic phenotypes in 3bKO and fl/fl mice under a high fat diet (HFD) challenge and a cold challenge, and examined the underlying mechanisms mediating DNMT3b’s regulation of brown fat thermogenic program.

## 2. Materials and Methods

### 2.1. Mice

Mice with *Dnmt3b* knockout in brown fat-skeletal muscle lineage cells were generated by crossing Dnmt3b-floxed mice (fl/fl) (Mutant Mouse Regional Resource Centers (MMRRC, stock #029887, MMRRC at University of North Carolina, Chapel Hill, NC, USA) with *Myf5-Cre* mice (Jackson Laboratory, Bar Harbor, Maine, USA, Stock #007893) [29] (*Myf5-Cre::Dnmt3b-fl/fl*, or 3bKO), where *Cre* is expressed in brown fat and skeletal lineage precursor cells under the control of the *Myf5* promoter [30]. The *Dnmt3b* allele in the floxed mouse was genetically modified by two loxP site insertion that flanks exons 16–19 encoding the catalytic motif [31]. The *Dnmt3b*-floxed mice have been backcrossed to the C57/BL6J background for more than five generations in our lab.

### 2.2. Metabolic Measurement

All animal procedures were approved by the Institutional Animal Care and Use Committee (IACUC) at Georgia State University (GSU). All mice were housed in a temperature (22 °C)- and humidity-controlled facility at GSU animal facility with a 12 h/12 h light–dark cycle and free access to water and food. For HFD feeding experiments, both female and male 3bKO mice and their fl/fl littermate controls were put on a HFD (Research Diets, New Brunswick, NJ, USA, D12492, 60% calorie from fat) for up to 20 weeks. Body weight of mice was measured weekly. Food intake was monitored in the TSE metabolic cage system, as described below, or measured in the single-housed animals over at least 5 consecutive days. A Minispec NMR body composition analyzer (Bruker BioSpin Corporation; Billerica, MA, USA) was used to determine the body composition of fat and lean mass. A PhenoMaster metabolic cage system (TSE Systems, Chesterfield, MO, USA) was used to measure oxygen consumption, carbon dioxide production, respiratory exchange ratio, locomotor activity and food/drink intake. Blood glucose levels were measured by OneTouch Ultra Glucose meter (LifeScan, Milpitas, CA, USA). Glucose tolerance and insulin sensitivity were assessed by glucose tolerance and insulin tolerance tests (GTTs and ITTs, respectively) as we previously described [32]. At the end of the experiments, tissues including fat pads, liver and skeletal muscle were dissected and collected for analysis of gene expression, protein expression and immunohistochemistry (IHC). Blood was collected to analyze plasma lipid and cholesterol profiles as we previously described [33]. Briefly, total cholesterol (TC) (Pointe Scientific, Canton, MI, USA, c7510-120), free cholesterol (FC) (Wako, Richmond, VA, USA, 993-02501), and triglyceride (TG) (Wako 998-02992 and 992-02892) concentrations were measured using enzymatic assays according to the manufacturer’s instructions.

### 2.3. Quantitative RT-PCR Analysis of mRNA

Quantitative PCR analysis was conducted as we previously described [26,32]. Briefly, total RNA from adipose tissue or skeletal muscle was isolated using a Tri Reagent kit (Molecular Research Center, Cincinnati, OH, USA). The mRNA levels of genes of interest were measured using an Applied Biosystems QuantStudio 3 real-time PCR system (ThermoFisher Scientific, Waltham, MA, USA) with a TaqMan Universal PCR Master Mix kit (ThermoFisher Scientific) as we previously described [34]. The sequences of the primer and probe pairs used in the assays are as follows. UCP1: Forward 5′-CACCTTCCCGCTGGACACT-3′; Reverse 5′-CCCTAGGACACCTTTATACCTAATGG-3′; Probe 5′-AGCCTGGCCTTCACCTTGGATCTGA-3′. Cyclophilin: Forward 5′-GGTGGAGAGCACCAAGACAGA-3′; Reverse 5′-GCCGGAGTCGACAATGATG-3′; Probe 5′-ATCCTTCAGTGGCTTGTCCCGGCT-3′. Other TaqMan primers/probes for all the genes measured were purchased from Applied Biosystems (ThermoFisher Scientific) as listed in Supplementary Table S1. All gene expression data were normalized to cyclophilin.

### 2.4. Immunoblotting

Immunoblotting for protein detection was conducted as we described [27,32]. Fat tissues were homogenized in a modified radioimmunoprecipitation assay (RIPA) lysis buffer supplemented with 1% protease inhibitor mixture and 1% phosphatase inhibitor mixture (Sigma-Aldrich, St. Louis, MO, USA). Tissue lysates were resolved by SDS-PAGE gels. Proteins on the gels were transferred to nitrocellulose membranes (Bio-Rad, Hercules, CA, USA), which were then blocked, washed, and incubated with various primary antibodies, followed by Alexa Fluor 680-conjugated secondary antibodies (ThermoFisher Scientific). The blots were developed with a Li-COR Imager System (Li-COR Biosciences, Lincoln, NE, USA). Primary antibodies used were as follows: Anti-UCP1 antibody (1:500, ab23841, Abcam, Cambridge, MA, USA); Anti-α-Tubulin antibody (1:1000, ABCENT4777, Advanced BioChemicals, Lawrenceville, GA, USA); Mitochondrial total OXPHOS protein antibody set (Abcam,ab110413); and Anti-pHSL (4126s, Cell Signaling Technology, Danvers, MA, USA); DNMT3b (sc-393845, Santa Cruz, Dallas, TX, USA, sc-393845); HSL (Cell Signaling, 4107s).

### 2.5. Immunohistochemistry (IHC)

IHC staining of UCP1 was conducted as we described [34,35]. Briefly, adipose tissues were fixed in 10% neutral formalin, embedded in paraffin and sectioned, followed by processing for hematoxylin and eosin (H&E) staining or immuno-staining with a UCP1 antibody (1:150, Abcam, ab10983) as we previously described [34,35]. Histology images were captured using a Nikon Eclipse E800 Microscope.

### 2.6. RNA-Sequencing Analysis

Total RNA was extracted and was commercially sequenced by Beijing Genomics Institute (BGI, Shenzhen, Guangdong, China). According to the report from BGI, after total RNA extraction and digestion with DNase I, mRNA was enriched with the oligo(dT) magnetic beads, fragmented (about 200 bp), and used for cDNA synthesis with random hexamer-primer. The double-stranded cDNA was ligated with sequencing adaptors and PCR amplified. RNA-seq libraries were then generated for sequencing with Illumina HiSeqTM 2000 (SE50). For quality control, RNA and library preparation integrity were verified using Agilent 2100 BioAnalyzer system and ABI StepOnePlus Real-Time PCR System.

For bioinformatics analysis, raw reads were filtered to remove adaptor sequences and low quality data. Filtered clean reads were mapped to reference sequences (University of California Santa Cruz Mouse Genome Browser mm9 Assembly) using SOAPaligner/SOAP2 [36]. Reads Per kilobase per Million reads (RPKM) were calculated to represent the gene expression level, and were used for comparing differentially expressed genes (DEGs) among groups identified as presenting more than two-fold increase or more than 50% decrease, and false discovery rate (FDR) < 0.001. DEGs were further used for Gene Ontology (GO) enrichment analysis, pathway enrichment analysis, cluster analysis, protein–protein interaction network analysis and transcription factor analysis. Heatmaps and volcano plots representing the differentially expressed genes were generated by using ComplexHeatmap v2.0.0 in R3.6.0 and EnhancedVolcano v1.2.0 respectively as previously described [37]. The RNA-seq data has been deposited into Gene Expression Omnibus (GEO) and assigned a number as GSE173765.

### 2.7. Pyrosequencing Analysis of the Mef2 Promoter

The pyrosequencing analysis was conducted as we previously described [32,38]. Briefly, genomic DNA was isolated from iBAT by phenol-chloroform extraction, followed by bisulfite conversion using an EpiTech Bisulfite Kit (Qiagen, Valencia, CA, USA, 59104). The DNA fragments covering bisulfite-converted CpG sites at the Mef2 promoter was PCR-amplified and the PCR products were sent to EpiGenDx (Hopkinton, MA, USA) for pyrosequencing. The sequences of PCR and pyrosequencing primers were listed in Supplementary Table S2.

### 2.8. Statistics

Data were expressed as mean ± SEM. Data collected from different groups in the experiments were analyzed by using Prism 7.0 (GraphPad Software Inc., San Diego, CA, USA). Unpaired Student’s *t* test was used to test statistical significance between two groups while one-way analysis of variance (ANOVA) was used to compare three or more groups. Statistical significance is considered at *p* < 0.05. Both female and male mice were used in this study. Age-matched littermate fl/fl mice were used as controls. Statistical significance between the different genotypes was tested for each diet, sex and the same age of the animals. Repeated-measures ANOVAs were performed on data from the TSE metabolic cage experiments, GTTs and ITTs, and weekly body weight. Follow-up specific tests were performed on the GTT and ITT data using a Bonferroni correction if the main effect was relevant.

## 3. Results

### 3.1. Dnmt3b Deficiency Promotes Diet-Induced Obesity

**Figure 1:**
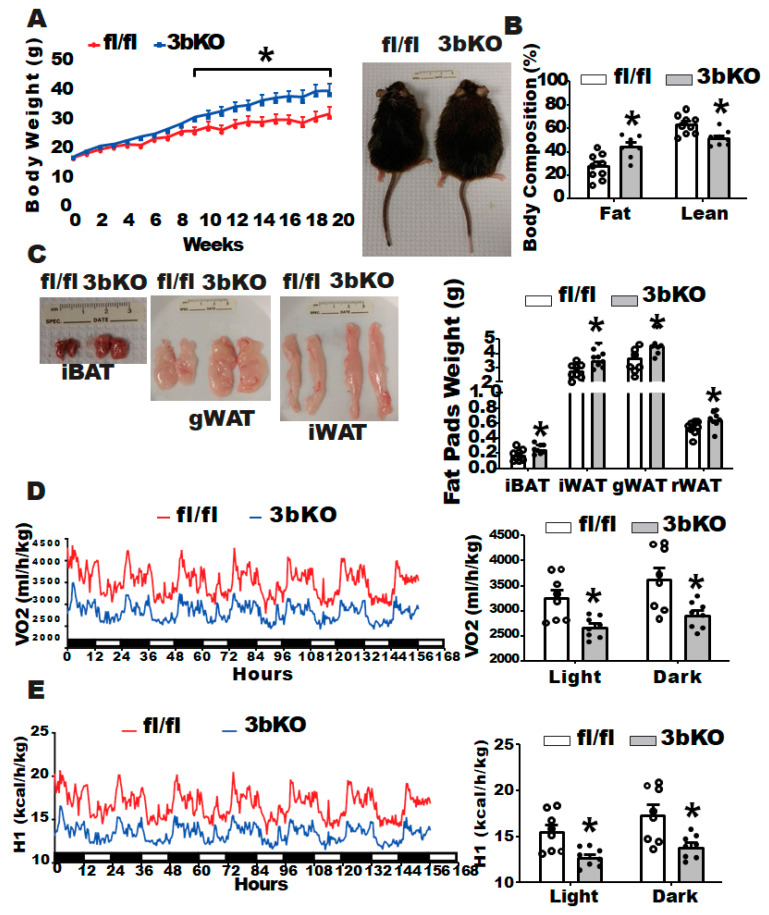
*Dnmt3b* deficiency in brown fat promotes high fat diet (HFD)-induced obesity in female mice. Five-week old female 3bKO and their littermate control fl/fl mice were put on a HFD (Research Diets D12492, 60% calorie from fat) for 20 weeks and were maintained at room temperature (22 °C) throughout the experiment. (**A**) Body weight growth curve in female 3bKO and fl/fl mice fed HFD. (**B**) Body composition measured by a Bruker NMR body composition analyzer in the female 3bKO and fl/fl mice fed HFD. (**C**) Fat pad weight of interscapular brown adipose tissue (iBAT), inguinal white adipose tissue (iWAT), gonadal WAT (gWAT)) and retroperitoneal WAT (rWAT) in the female 3bKO and fl/fl mice fed HFD. (**D**) Oxygen consumption measured by TSE PhenoMaster metabolic cage systems in 16-week old female 3bKO and fl/fl mice fed HFD. (**E**) Calculated energy expenditure based on the oxygen consumption in (**D**). All data are expressed as mean ± SEM; *n* = 8/group; * *p* < 0.05 vs. fl/fl.

**Figure 2:**
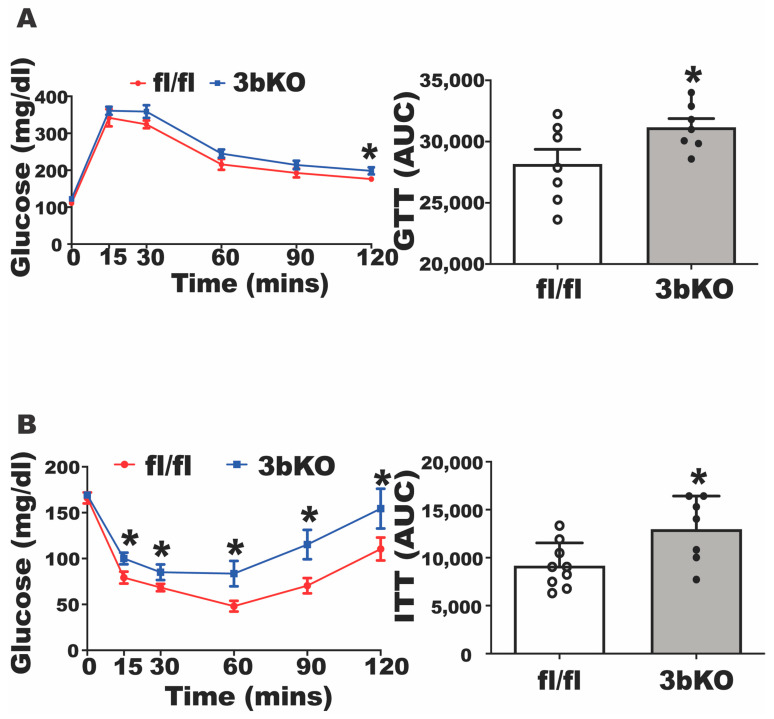
*Dnmt3b* deficiency in brown fat exacerbates HFD-induced insulin resistance in female mice. Five-week old female 3bKO and their littermate control fl/fl mice were put on HFD for 20 weeks and were maintained at room temperature (22 °C) throughout the experiment. (**A**) Glucose tolerance test (GTT) in 18-week old female 3bKO and fl/fl mice fed HFD. (**B**) Insulin tolerance test (ITT) in 20-week old female 3bKO and fl/fl mice fed HFD. All data are expressed as mean ± SEM; *n* = 7/group; * *p* < 0.05 vs. fl/fl.

To determine the role of brown fat Dnmt3b in the regulation of diet-induced obesity, we generated a genetic model with deletion of Dnmt3b in brown fat-skeletal muscle lineage precursor cells (3bKO) by crossing Dnmt3b-floxed (fl/fl) mice with Myf5-cre mice. The PCR genotyping that indicates the correct genotypes for the 3bKO mice and their fl/fl littermates is shown in Supplementary Figure S1A. Several lineage tracing studies show that brown fat and skeletal muscle share the same developmental origins [30,39]. Since the somitic Myf5-expressing multipotent progenitor cells can develop into brown adipocytes [40], Myf5 Cre line has been used to generate models with early deletion of genes of interest for the study of brown fat development and function [30,41,42]. As expected, 3bKO mice had a 53% reduction in Dnmt3b mRNA expression in brown fat (Supplementary Figure S1B). Immunoblotting analysis also showed a significant reduction in DNMT3b protein levels in interscapular brown fat (iBAT) and gastrocnemius skeletal muscle (Supplementary Figure S1C,D) but not in inguinal white adipose tissue (iWAT), gonadal white adipose tissue (gWAT) (Supplementary Figure S1E,F) and liver (Supplementary Figure S1G) of the 3bKO mice. We did not find any difference in body weight and body fat composition between 4-month old female 3bKO mice and their littermate fl/fl controls fed a regular chow diet (Supplementary Figure S2). We then put the female 3bKO mice on HFD and conducted metabolic characterization. The female 3bKO mice on HFD still showed reduced DNMT3b protein levels in iBAT (Supplementary Figure S3A), albeit to a lesser extent compared to chow-fed mice, possibly due to cell composition change in iBAT by HFD feeding, but not in other fat depots (iWAT and gWAT) and liver (Supplementary Figure S3B–D). We found that the female 3bKO mice gained significantly more weight compared to their fl/fl littermates along the course of HFD feeding (Figure 1A). NMR body composition analysis revealed higher fat mass composition with a corresponding lower lean mass composition in the 3bKO mice than those of the control mice (Figure 1B). In support of increased adiposity, the female 3bKO mice also exhibited an increased weight of various fat pads including interscapular WAT (iBAT), inguinal WAT (iWAT), gonadal WAT (gWAT) and retroperitoneal WAT (rWAT) (Figure 1C). We used a PhenoMaster metabolic cage system to characterize the energy metabolism and found that the female 3bKO mice exhibited lower oxygen consumption and energy expenditure (Figure 1D,E), which may largely account for increased body weight and adiposity in these female mice, as there were no differences in respiratory exchange ratio (RER), locomotor activity and food intake between the 3bKO and their fl/fl littermate control mice (Supplementary Figure S4A–C). With increased adiposity, the female 3bKO mice displayed significantly impaired insulin sensitivity as shown by mild glucose intolerance in GTT and more severe insulin resistance in ITT tests when compared to fl/fl mice (Figure 2A,B). However, we did not observe any changes in triglyceride content, total and free cholesterol levels in the liver and blood of the 3bKO mice (Supplementary Figure S5). These data indicate that Dnmt3b deletion in Myf5^+^ brown fat progenitor cells promotes diet-induced obesity and insulin resistance.

### 3.2. Dnmt3b Deficiency Down-Regulates Thermogenic Program in Brown Fat

**Figure 3:**
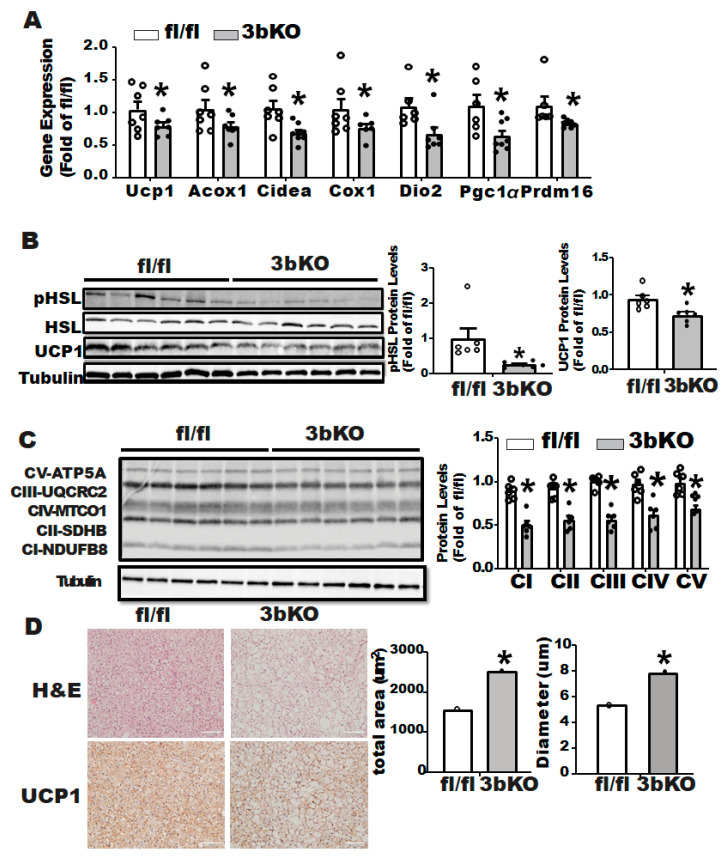
*Dnmt3b* deficiency impairs brown fat thermogenic program in HFD-fed female mice. Five-week old female 3bKO and their littermate control fl/fl mice were put on HFD for 20 weeks. (**A**) Thermogenic gene expression in the iBAT measured by quantitative RT-PCR in the female 3bKO and fl/fl mice fed HFD. (**B**) Immunoblotting of UCP1 and phosphor-HSL. (**C**) and mitochondrial respiratory chain complex proteins in the iBAT of the female 3bKO and fl/fl mice fed HFD. (**D**) H&E staining and Immunohistochemical (IHC) staining of UCP1 (images on left panel) and quantitation of brown adipocyte diameter and area (bar graphs on right panel) in the iBAT of the female 3bKO and fl/fl mice fed HFD. All data are expressed as mean ± SEM; *n* = 6/group; * *p* < 0.05 vs. fl/fl.

We further characterized the thermogenic program in iBAT of the female 3bKO mice. Quantitative RT-PCR analysis revealed a decreased expression of thermogenic genes such as *Ucp1*, acyl-CoA oxidase 1 (*Acox1*), cell death-inducing DNA fragmentation factor alpha subunit-like effector A (*Cidea*), cytochrome C oxidase subunit 1 (*Cox1*), type 2 diodinase (*Dio2*), peroxisome proliferative activated receptor γ coactivator 1α (*Pgc1α*) and PR domain-containing 16 (*Prdm16)* in iBAT of 3bKO mice (Figure 3A). This was associated with a down-regulation of UCP1 protein levels as well as phosphor-hormone sensitive lipase (pHSL) levels in iBAT of the 3bKO mice, as measured by immunoblotting (Figure 3B). In addition, we found a down-regulation of proteins in the mitochondrial respiratory chain complexes in the iBAT of the 3bKO mice by immunoblotting, including NADH dehydrogenase 1β subcomplex 8 (NDUFB8) in complex I, succinate dehydrogenase complex subunit B (SDHB) in complex II, mitochondrial Cytochrome b-c1 complex subunit 2 (UQCRC2) in complex III, mitochondrially encoded cytochrome c oxidase I (MTCO1) in complex IV, and mitochondrial ATP synthase F1 subunit alpha (ATP5F1A) in complex V (Figure 3C). In consistence, immunohistochemical analysis revealed larger brown adipocytes (increased cell diameters and areas) with much less UCP1 staining in the iBAT of the 3bKO mice (Figure 3D).

### 3.3. Dnmt3b Deficiency Induces Myogenesis in Brown Fat

**Figure 4:**
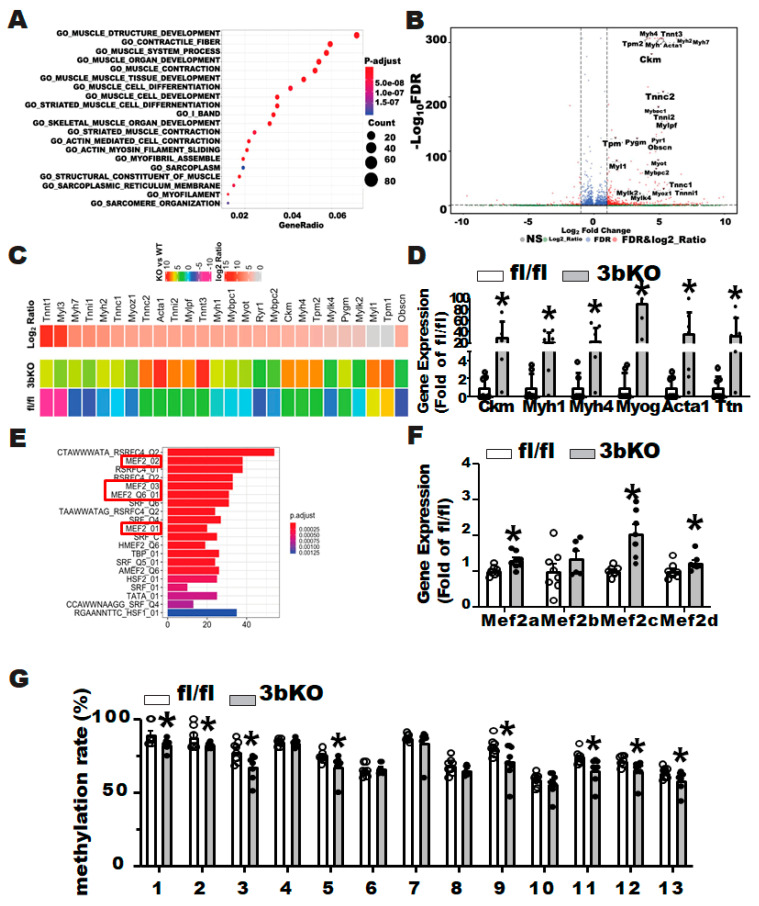
*Dnmt3b* deficiency induces myogenesis in brown fat. RNA-seq analysis was conducted using the iBAT of 25-week old female 3bKO and fl/fl mice fed HFD. (**A**) Bioinformatics pathway analysis. (**B**) The volcano plot of differentially expressed genes in the iBAT of the female 3bKO vs. fl/fl mice. (**C**) The heatmap of myogenic marker gene expression in the iBAT of the female 3bKO vs. fl/fl mice. (**D**) Quantitative PCR analysis of myogenic markers in the iBAT of the female 3bKO and fl/fl mice. (**E**) Motif enrichment analysis of the myogenic gene promoters. (**F**) Quantitative PCR analysis of *Mef2* family members in the iBAT of the female 3bKO and fl/fl mice. (**G**) Pyrosequencing analysis of the *Mef2c* promoter in the iBAT of the female 3bKO and fl/fl mice. All data are expressed as mean ± SEM; * *p* < 0.05 vs. fl/fl.

To determine the molecular mechanism whereby Dnmt3b deficiency promotes diet-induced obesity, we performed a RNA-seq analysis using brown fat from HFD-fed female 3bKO mice and control fl/fl mice to unbiasedly examine the gene expression profiles. We found that 628 genes were differentially regulated by Dnmt3b deficiency (Log_2_ fold change ≥ 0.5 or ≤−0.5). Among these genes, 512 genes were up-regulated, whereas 116 genes were down-regulated by *Dnmt3b* deficiency. Surprisingly, a pathway analysis disclosed a significant up-regulation of myogenic pathways, including genes involved in muscle development, structure and contraction, in *Dnmt3b*-deficient brown fat (Figure 4A and Supplementary Figure S6). Indeed, a volcano plot revealed an up-regulation of a panel of myogenic genes in *Dnmt3b*-deficient iBAT (Figure 4B). This was consistent with a hierarchical cluster analysis indicating a broad up-regulation of myogenic genes (Figure 4C). Quantitative RT PCR analysis further confirmed the induction of myogenic markers, such as muscle creatine kinase (*Ckm*), myosin heavy chain polypeptide 1 (*Myh1*), myosin heavy chain polypeptide 4 (*Myh4*), myogenin (*Myog*), skeletal muscle actin alpha 1 (*Acta1*) and Titin (*Ttn*), in iBAT of 3bKO mice (Figure 4D). Further motif enrichment analysis on up-regulated myogenic gene promoters identified myocyte enhancer factor 2 (MEF2) binding motif ranked among top candidates (Figure 4E). The transcriptional factor MEF2 family consists of four members: MEF2A, 2B, 2C and 2D, which play diverse but redundant roles in the development of various cell types including muscles (skeletal, cardiac, and smooth), neurons and hematopoietic and immune cells [43]. We found that three out of four MEF2 members including *Mef2a*, *2c*, and *2d* exhibited increased expression in *Dnmt3b*-deficient iBAT (Figure 4F). Since *Mef2c* expression was most up-regulated by *Dnmt3b* deficiency in BAT, we assessed whether *Dnmt3b* regulates *Mef2c* expression by modulating *Mef2c* promoter DNA methylation. Indeed, pyrosequencing analysis revealed decreased DNA methylation at 8 out of 13 CpG sites at *Mef2c* promoter and 5′ region in iBAT of 3bKO mice (Figure 4G, and Supplementary Figure S7), which may explain the up-regulated *Mef2c* expression in *Dnmt3b*-deficient iBAT.

Since Myf5^+^-precursor cells could develop into brown adipocyte and skeletal muscle cells, we examined whether Dnmt3b deletion in Myf5^+^-lineage precursor cells altered skeletal muscle development. As expected, there was around 55% reduction in *Dnmt3b* mRNA level in gastrocnemius muscle of the female 3bKO mice compared to fl/fl mice (Supplementary Figure S8A). A similar reduction in DNMT3b protein levels was also observed in the muscle of the 3bKO mice (Supplementary Figure S8B). However, further quantitative RT-PCR analysis did not show any differences in myogenic marker expression and brown fat thermogenic gene expression in gastrocnemius muscle between female 3bKO and fl/fl mice (Supplementary Figure S8C,D), suggesting that *Dnmt3b* deletion does not affect skeletal muscle development or cause brown fat lineage switch. To further assess the potential alteration of energy metabolism in the skeletal muscle of knockout mice, we examined the protein levels of mitochondrial respiratory chain complexes, but still found no changes (Supplementary Figure S8E). In sum, these data indicate that *Dnmt3b* deficiency causes brown fat remodeling by induction of myocyte-like brown adipocyte formation.

### 3.4. Dnmt3b Deficiency Does Not Change Body Weight in Male Mice

We also measured body weight of the male 3bKO mice fed regular chow or HFD. Similar to the female 3bKO mice, 3-month old male 3bKO mice fed regular chow diet did not show any changes in body weight and body fat composition (Supplementary Figure S9A,B). Unlike the female 3bKO mice, the male 3bKO mice fed HFD did not show body weight difference compared to control fl/fl mice (Supplementary Figure S9C). In addition, there was no difference in body fat composition and individual fat pad mass (albeit a tendency toward higher in the 3bKO mice) between the male 3bKO mice on HFD and the control fl/fl mice, whereas lean mass was slightly reduced in the male 3bKO mice on HFD compared to the fl/fl mice (Supplementary Figure S9D,E).

### 3.5. Dnmt3b Deficiency Suppresses Thermogenic Program in Brown Fat of the Female 3bKO Mice

**Figure 5:**
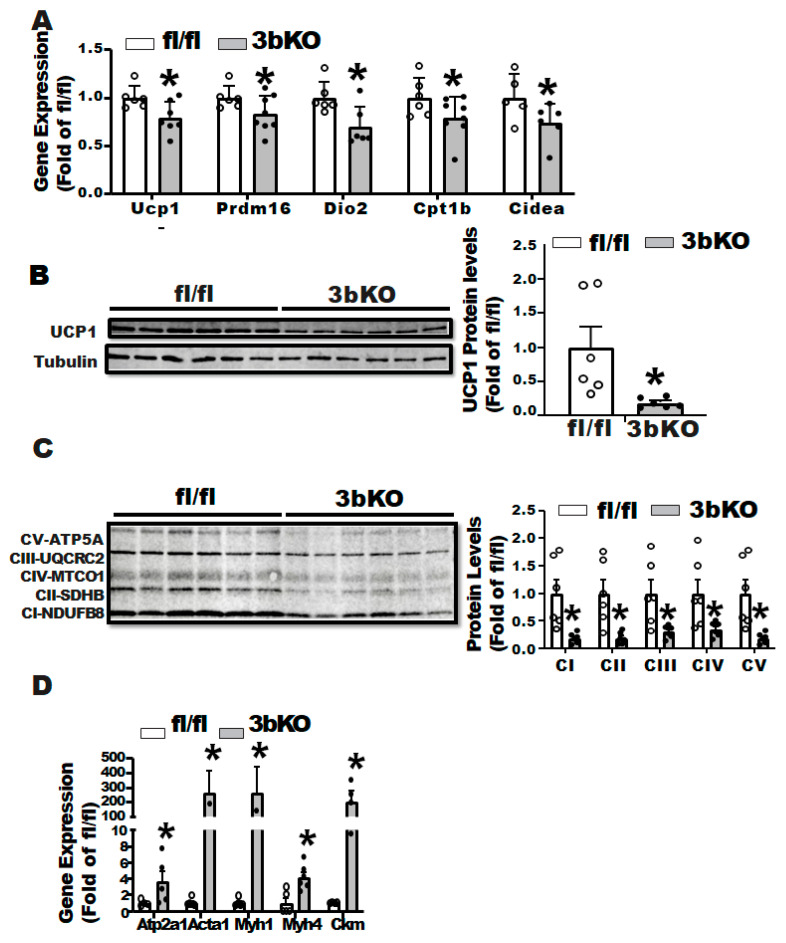
*Dnmt3b* deficiency impairs brown fat thermogenic program in cold-challenged female mice. Four-month old female 3bKO and their littermate control fl/fl mice were challenged with cold at 5 °C for 7 days. (**A**) Quantitative RT-PCR analysis of thermogenic gene expression in the iBAT. (**B**) Immunoblotting of UCP1 and (**C**) mitochondrial respiratory chain complex proteins. (**D**) Quantitative RT-PCR analysis of myogenic gene expression in the iBAT. All data are expressed as mean ± SEM; *n* = 6/group; * *p* < 0.05 vs. fl/fl.

Cold and diet are the two primary triggers that induce brown fat thermogenesis. To determine the role of Dnmt3b in regulating cold-induced thermogenesis, we subjected the female 3bKO and their littermate control fl/fl mice to a cold challenge. After a 7-day cold exposure, there were no differences in body weight, body fat composition and fat pad weight between the 3bKO and their littermate controls (Supplementary Figure S10A–C). However, the 3bKO mice displayed a decreased expression of thermogenic genes such as Ucp1, Prdm16, Dio2 etc in iBAT (Figure 5A). This was associated with a down-regulation of UCP1 protein levels as well as proteins in the mitochondrial respiratory chain complexes including NDUFB8, SDHB, UQCRC2, MTCO1, ATP5F1A in iBAT of the 3bKO mice by immunoblotting (Figure 5B,C). Interestingly, we also discovered a dramatic induction of myogenic markers, such as Atp2a1, Acta1, Myh1, Myh4, and Ckm in iBAT of the 3bKO mice (Figure 5D). The reciprocal down-regulation of thermogenic gene expression and up-regulation of myogenic gene expression may suggest a brown adipocyte to myocyte remodeling, leading to the impaired cold-induced thermogenesis in the female 3bKO mice.

We also conducted a cold challenge experiment on the male 3bKO mice. Unlike the female 3bKO mice, the male 3bKO mice did not show any changes in thermogenic gene expression and UCP1 protein levels in iBAT, although they did have increased myogenic gene expression in iBAT (Supplementary Figure S11A–C). These data suggest a sexual dimorphism in the effect of Dnmt3b in the regulation of brown fat thermogenesis.

## 4. Discussion

In this study, we have generated a genetic model with *Dnmt3b* knockout in *Myf5*^+^-brown fat-skeletal lineage precursor cells (3bKO mice). Female 3bKO mice display decreased energy expenditure and are prone to diet-induced obesity and insulin resistance. *Dnmt3b* deficiency in *Myf5*^+^-cells induces myogenic remodeling in brown fat, which may contribute to the dysregulation of energy metabolism in the knockout mice. The plausibility of this study was derived from prior observations that epigenetic regulation plays a key role in the development of obesity and its related diseases. We recently have reported the involvement of DNA methylation in the regulation of several metabolic pathways. [24,25,32,44,45,46]. On the other hand, several lines of evidence have suggested a role for DNA methylation in the regulation of brown fat thermogenesis. For example both *Ucp1*, the key thermogenic protein in brown fat, and *Pgc1α*, the master regulator of mitochondrial biogenesis, are subjected to DNA methylation modifications [47,48,49]. We therefore have been interested in understanding the role of DNA methylation in brown fat development and thermogenic function [28]. In consistence with our prior findings on *Dnmt1* or *3a*, deletion of *Dnmt3b* at early stage of brown fat development using *Myf5*-Cre also promoted diet-induced obesity and insulin resistance, which was associated with the induction of myogenic program in brown fat. Lineage tracing studies have provided strong evidence to support that brown fat and skeletal muscle share the same developmental origins [30,39]. Most brown adipocytes originate from a mesodermal progenitor population in the somites that also gives rise to skeletal myocytes [30,39]. The somitic multipotent progenitor cells featured by expression of transcriptional factors paired box 7 (*Pax7)*, engrailed 1 (*En1)* and *Myf5* can either develop into brown adipocytes through activation of *Prdm16*, early B cell factor 2 (*Ebf2)*, and zinc finger protein 516 (*Zfp516),* or commit to the skeletal myogenic pathway via activation of myogenic differentiation 1 (*Myod1)*, *Myog* and myogenic regulatory factor 4 (*Mrf4)* [50]. We found that the myogenic remodeling in brown fat due to Dnmt1 deficiency might be due to induction of *Myod1*, a master regulator of myogenesis, by its promoter demethylation [28]. Although the brown fat *Dnmt3b* knockout model in this study and *Dnmt1/3a* knockout models reported above share striking similarity in myogenic remodeling in brown fat, the mechanism underlying the myogenic switch in brown fat might be different. While we did not observe a significant change in *Myod1* expression from RNA-seq analysis in *Dnmt3b*-deficient brown fat, we, through motif enrichment analysis, identified MEF2 family transcriptional factors particularly Mef2a, 2c and 2d, whose expression is up-regulated in *Dnmt3b*-deficient brown fat. While all three MEF2s including 2A, 2C and 2D are involved in the development, morphogenesis and maintenance of various types of muscles including skeletal, cardiac and smooth muscle, each transcriptional factor has its own specialty in doing so [43]. Although MEF2A and 2C regulate skeletal muscle development and smooth muscle cell differentiation, respectively, the two transcriptional factors share most similarity in sequences and have overlapping and yet diverse functions in shaping skeletal muscle identity [43,51]. Since *Mef2c* expression is most up-regulated in *Dnmt3b*-deficient brown fat and has been shown to be regulated by DNA methylation [52], we assessed DNA methylation status at the *Mef2c* promoter and found *Dnmt3b* deficiency down-regulated DNA methylation levels at the *Mef2c* promoter, which may be responsible for the increased *Mef2c* expression in iBAT of 3bKO mice. Indeed, a prior report demonstrated that the Mef2c promoter activity is regulated by DNA methylation status [53]. The authors examined fully methylated vs. unmethylated Mef2c promoter activity and showed that luciferase activity of unmethylated Mef2c promoter was significantly higher than that of fully methylated Mef2c promoter [53]. However, we cannot rule out that *Dnmt3b* deficiency may also cause alterations of DNA methylation on the promoters of *Mef2a* and/or *Mef2d*.

Since Myf5^+^-precursor cells could develop into both brown adipocytes and skeletal muscle cells, employing Myf5^+^ Cre line inevitably inhibits Dnmt3b expression in skeletal muscle, as we showed in Supplementary Figures S1D and S8A,B. Skeletal muscle, which accounts for 40% of total body mass and 30% of resting metabolic rate in non-obese humans, plays a key role in the regulation energy metabolism and insulin stimulated glucose disposal [54]. Given the importance of skeletal muscle in overall energy metabolism and glucose homeostasis, we examined the expression of myogenic markers that represent skeletal muscle development and function. The myogenic gene expression does not show any difference between 3bKO mice and their littermate controls, nor does thermogenic gene expression in the muscle. We further assessed the protein levels of mitochondrial respiratory chain complexes in the gastrocnemius of the 3bKO mice but found no changes. Although these data suggest that Dnmt3b deficiency does not alter myogenic gene expression and the mitochondrial machinery, we cannot rule out functional changes in the skeletal muscle in the energy metabolism and glucose uptake. A more thorough assessment on skeletal muscle energy and nutrient metabolism would be required to determine its exact contribution. For instance, we have observed insulin resistance and glucose intolerance in ITTs and GTTs in the 3bKO mice. However, it is not clear whether the insulin resistance that occurs in the 3bKO mice is derived from the direct effect of muscle insulin resistance or secondary effect from the obese phenotype of the knockout mice. A hyperinsulinemic-euglycemic clamp experiment on the knockout mice before their body weight changes become evident would help distinguish the direct contribution of the skeletal muscle to the systemic insulin resistance.

Prior lineage chasing studies revealed the contribution of the Myf5 positive progenitor cells to the development of subcutaneous WAT [55], suggesting that Myf5 Cre may also knock down Dnmt3b in a portion of adipocytes residing within subcutaneous WAT. Although we did not find a significant reduction in Dnmt3b mRNA expression in subcutaneous WAT of 3bKO mice, we cannot rule out that some Myf5 originated adipocytes, albeit at a lower number, may have Dnmt3b deletion. How the Dnmt3b deletion in iWAT may contribute to the energy metabolism and obese phenotype of 3bKO mice is not clear. Recent studies discovered a subset of glycolytic beige adipocytes featured by a myogenic state [56]. It is not clear, however, whether Dnmt3b deletion in Myf5 lineage adipocytes would affect the formation of glycolytic beige adipocytes, contributing to the decreased energy expenditure and increased obesity observed in 3bKO mice.

Unlike the female 3bKO mice, HFD-fed male 3bKO mice do not show any differences in body weight or fat mass when compared to that of fl/fl mice. Sexual dimorphism frequently occurs in metabolic phenotypes of both humans and rodents. For one, males and females have different fat composition and distribution in humans [57,58]. This might be due to differential lipid metabolism between the two genders [59]. Another potential mechanism may be attributed to the sex hormone estrogen and its receptors that have been shown to play a pivotal role in various metabolic pathways [60]. Future experiments involving ovariectomy may disclose the role of estrogen in the development of obesity in female 3bKO mice.

## 5. Conclusions

Our data show that *Dnmt3b* deficiency in Myf5^+^-brown fat precursor cells inhibits thermogenic program in brown fat, decreases energy expenditure, and promotes diet-induced obesity and insulin resistance in female mice. The inhibition of thermogenic function is associated with a myogenic remodeling in brown fat, which may result from increased *Mef2c* expression due to decreased DNA methylation at its promoter by *Dnmt3b* deficiency. Our data demonstrate that *Dnmt3b* plays an important role in the regulation of brown fat function, energy metabolism and obesity in female mice.

## Data Availability

All datasets will be available upon request to the corresponding authors Hang Shi and Bingzhong Xue.

## Supplementary Materials

The following are available online at https://www.mdpi.com/article/10.3390/biom11081087/s1, Figure S1: Generation of 3bKO mice, Figure S2: (A) Body weight (*n* = 12/group) and (B) body fat composition (*n* = 6/group) of 4-month old female 3bKO and their littermate fl/fl mice fed chow diet, Figure S3: Dnmt3b protein levels in iBAT (A), iWAT (B), gWAT (C) and liver (D) of 6-month old female 3bKO and fl/fl mice fed HFD for 20 weeks. Figure S4: Metabolic characterization of 16-week old female 3bKO and fl/fl control mice on HFD. 5-week old female 3bKO and their littermate control fl/fl mice were put on HFD for 20 weeks. Figure S5: Lipid profile in the liver and blood of 25-week old female 3bKO and fl/fl control mice on HFD. Figure S6: Bioinformatic pathway analysis of RNA-seq data using iBAT of 25-week old female 3bKO and fl/fl control mice on HFD. Figure S7: Schematic illustration of CpG sites at the Mef2c promoter. Figure S8: Quantitative RT-PCR analysis of Dnmt3b mRNA (A), immunoblotting of DNMT3b protein (B), quantitative RT-PCR analysis of skeletal muscle markers (C) and thermogenic genes (D), and immunoblotting of mitochondrial respiratory chain protein (E) in gastrocnemius skeletal muscle of 25-week old female 3bKO and fl/fl mice on HFD. Supplemental Figure S9: Dnmt3b deficiency does not change body weight in male mice fed chow diet. Figure S10: 4-month old female 3bKO and their littermate fl/fl mice were challenged with cold at 5 °C for 7 days. Figure S11: 4-month old male 3bKO and their littermate fl/fl mice were challenged with cold at 5 °C for 7 days.
