# Supplementary material for: Dnmt3b Deficiency in Myf5+-Brown Fat Precursor Cells Promotes Obesity in Female Mice
Source: Biomolecules. 2021 Jul 23;11(8):1087. doi: 10.3390/biom11081087 (PMC8393658; doi:10.3390/biom11081087)
Supplement: Supplementary file 1 [file biomolecules-11-01087-s001.zip › biomolecules-1297474-SI.pdf]

Supplemental figure legends

Supplemental Figure S1. Generation of 3bKO mice. (A) PCR genotyping of the Myf5 Cre allele and flox allele in the female 3bKO and their littermate fl/fl mice respectively. *Dnmt3b* mRNA levels in interscapular BAT (iBAT) (B), DNMT3b protein levels in iBAT (C), gastrocnemius skeletal muscle (D), gonadal white adipose tissue (gWAT) (E), inguinal WAT (iWAT) (F) and liver (G) of 8-week old female 3bKO and fl/fl mice. All data are expressed as mean ± SEM; n=5/group; *p<0.05 vs. fl/fl.

Supplemental Figure S2. (A) Body weight (n=12/group) and (B) body fat composition (n=6/group) of 4-month old female 3bKO and their littermate fl/fl mice fed chow diet. Body composition measured by a Bruker NMR body composition analyzer. All data are expressed as mean ± SEM.

Supplemental Figure S3. Dnmt3b protein levels in iBAT (A), iWAT (B), gWAT (C) and liver (D) of 6-month old female 3bKO and fl/fl mice fed HFD for 20 weeks. All data are expressed as mean ± SEM; n=6/group; *p<0.05 vs. fl/fl.

Supplemental Figure S4. Metabolic characterization of 16-week old female 3bKO and fl/fl control mice on HFD. 5-week old female 3bKO and their littermate control fl/fl mice were put on HFD for 20 weeks. Respiratory exchange ratio (RER) (A), locomotor activity (B), and food intake (C) measured by TSE PhenoMaster metabolic cage systems in the female 3bKO and fl/fl mice fed HFD. All data are expressed as mean ± SEM; n=8/group.

Supplemental Figure S5. Lipid profile in the liver and blood of 25-week old female 3bKO and fl/fl control mice on HFD. 5-week old female 3bKO and their littermate control fl/fl mice were put on HFD for 20 weeks. (A-C) Liver total triglyceride (TG) (A), total cholesterol (TC) (B), and free cholesterol (FC) (C). (D-F) Serum TG (D), TC (E), and FC (F). All data are expressed as mean ± SEM; n=6/group.

Supplemental Figure S6. Bioinformatic pathway analysis of RNA-seq data using iBAT of 25-week old female 3bKO and fl/fl control mice on HFD.

Supplemental Figure S7. Schematic illustration of CpG sites at the Mef2c promoter.

Supplemental Figure S8. Quantitative RT-PCR analysis of Dnmt3b mRNA (A), immunoblotting of DNMT3b protein (B), quantitative RT-PCR analysis of skeletal muscle markers (C) and thermogenic genes (D), and immunoblotting of mitochondrial respiratory chain protein (E) in gastrocnemius skeletal muscle of 25-week old female 3bKO and fl/fl mice on HFD. All data are expressed as mean ± SEM; n=6-8; *$p<0.05$ vs. fl/fl.

Supplemental Figure S9. Dnmt3b deficiency does not change body weight in male mice fed chow diet. (A) Body weight (n=6/group) and (B) body fat composition (n=6/group) of 3-month old male 3bKO and their littermate fl/fl mice fed chow diet. Dnmt3b deficiency does not change body weight in male mice fed HFD. 5-week old male 3bKO and their littermate control fl/fl mice were put on HFD for 18 weeks. (C) Body weight growth curve in male 3bKO and fl/fl mice fed HFD. (D) Body composition measured by a Bruker NMR body composition analyzer in 16-week old male 3bKO and fl/fl mice fed HFD. (E) Fat pad weight of interscapular brown adipose tissue (iBAT), inguinal white adipose tissue (iWAT) and epididymal WAT (eWAT)) in 25-week old male 3bKO and fl/fl mice fed HFD. All data are expressed as mean ± SEM; n=8-9/group.

Supplemental Figure S10. 4-month old female 3bKO and their littermate fl/fl mice were challenged with cold at 5 °C for 7 days. (A) Body weight, (B) body fat composition, and fat pad weight (C) of the female 3bKO and their littermate fl/fl mice after a 7-day cold exposure. iBAT: interscapular brown adipose tissue; iWAT: inguinal white adipose tissue; gWAT: gonadal white adipose tissue; rWAT: retroperitoneal white adipose tissue. All data are expressed as mean ± SEM; n=6-8/group.

Supplemental Figure S11. 4-month old male 3bKO and their littermate fl/fl mice were challenged with cold at 5 °C for 7 days. Quantitative RT-PCR analysis of thermogenic gene expression (A), immunoblotting of UCP1 protein (B) and quantitative RT-PCR analysis of myogenic gene expression (C) in the iBAT of the male 3bKO and their littermate fl/fl mice. All data are expressed as mean ± SEM; n=6-8/group.

Supplemental Figure S1

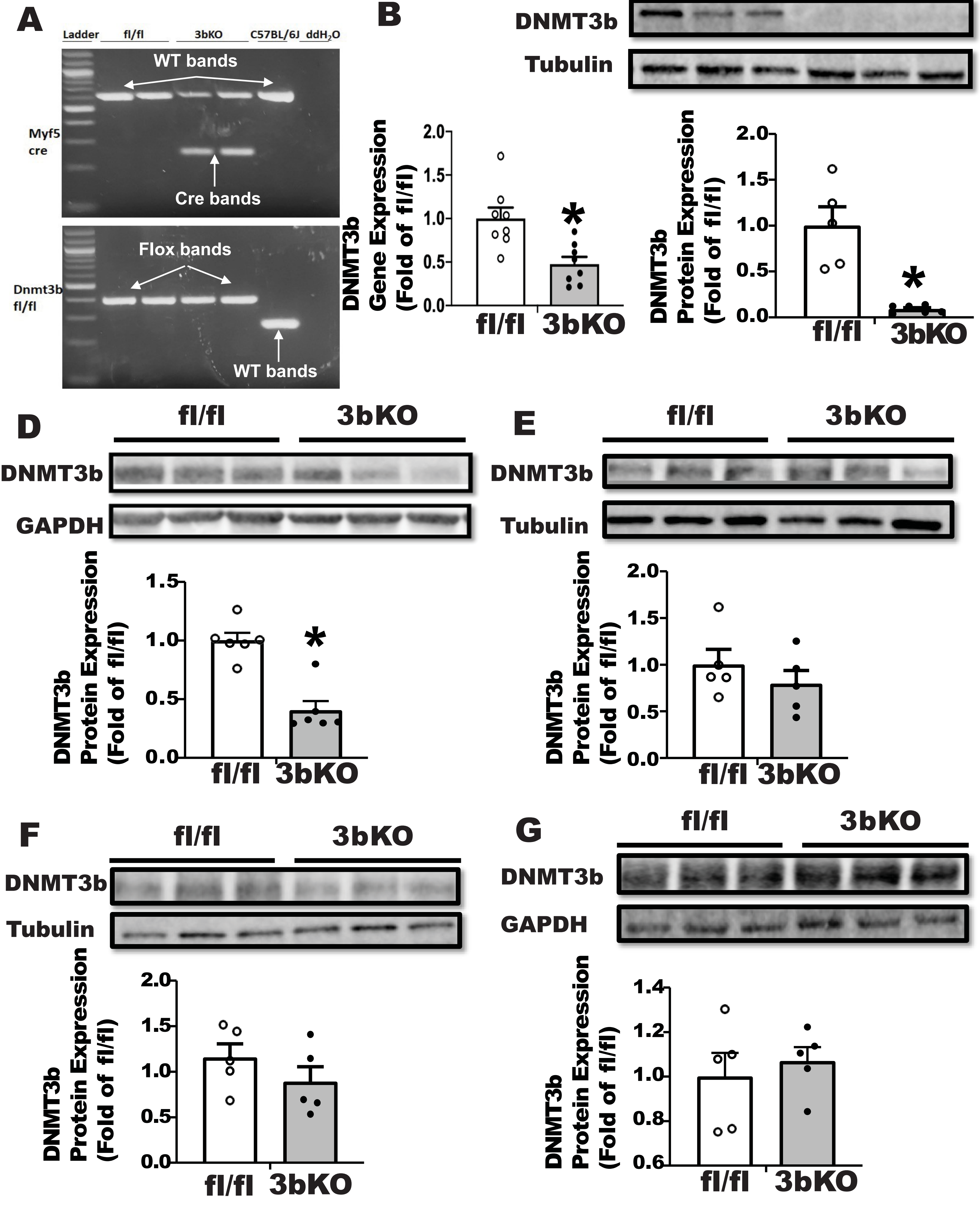

Supplemental Figure S2

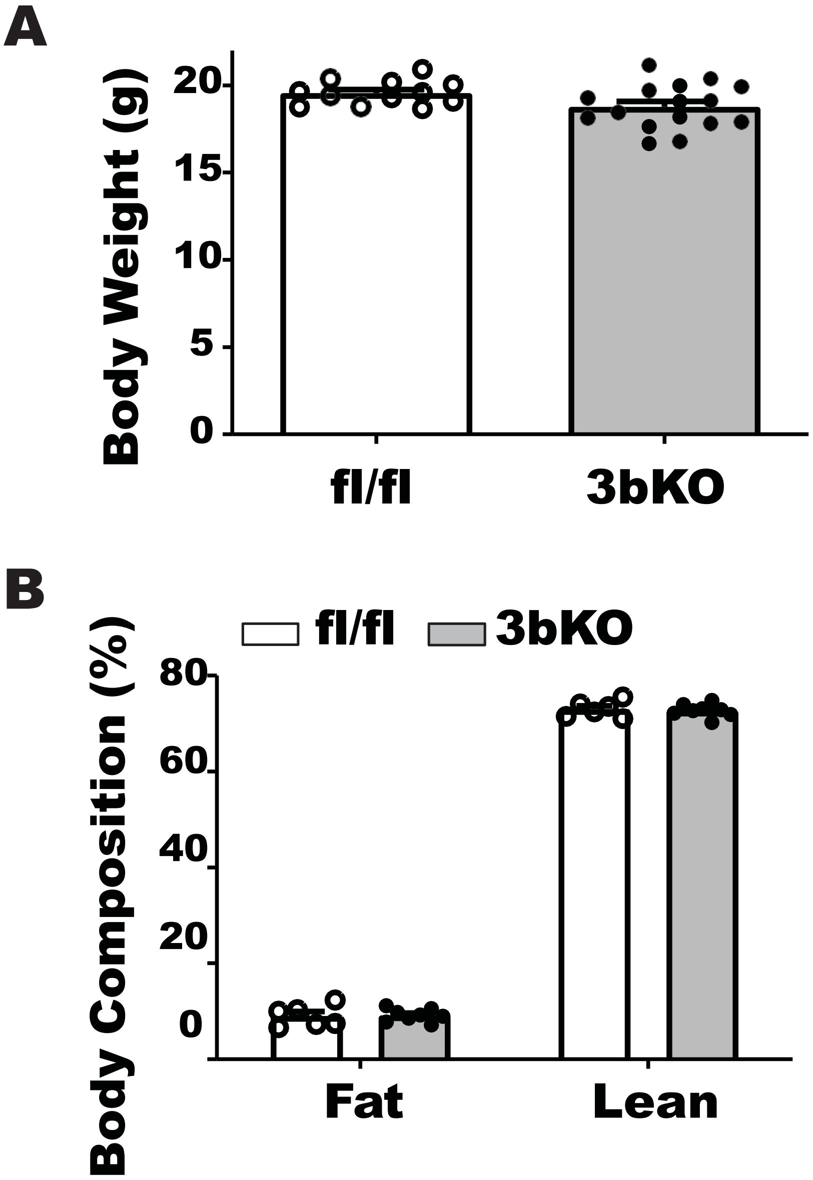

Supplemental Figure S3

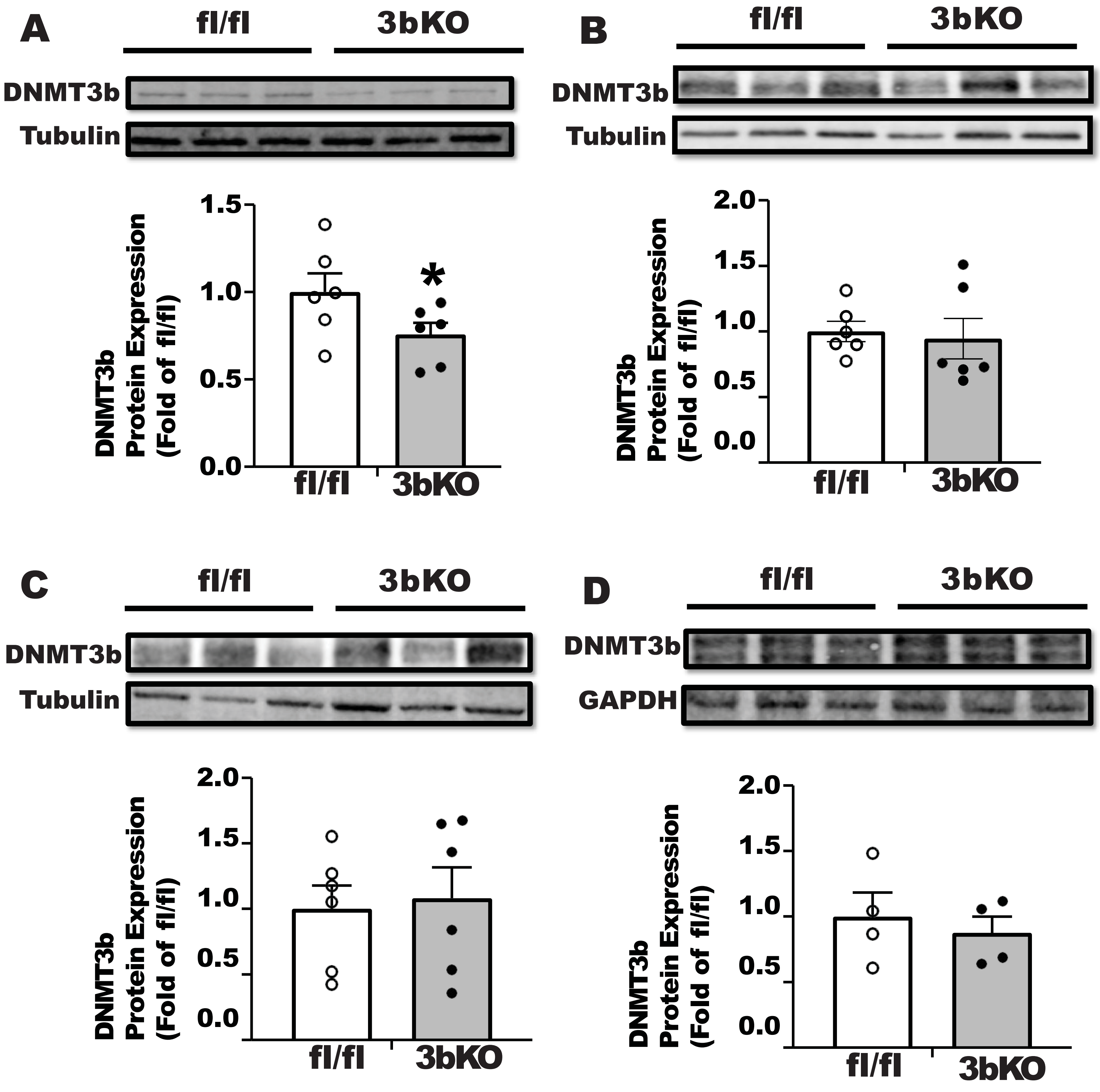

Supplemental Figure S4

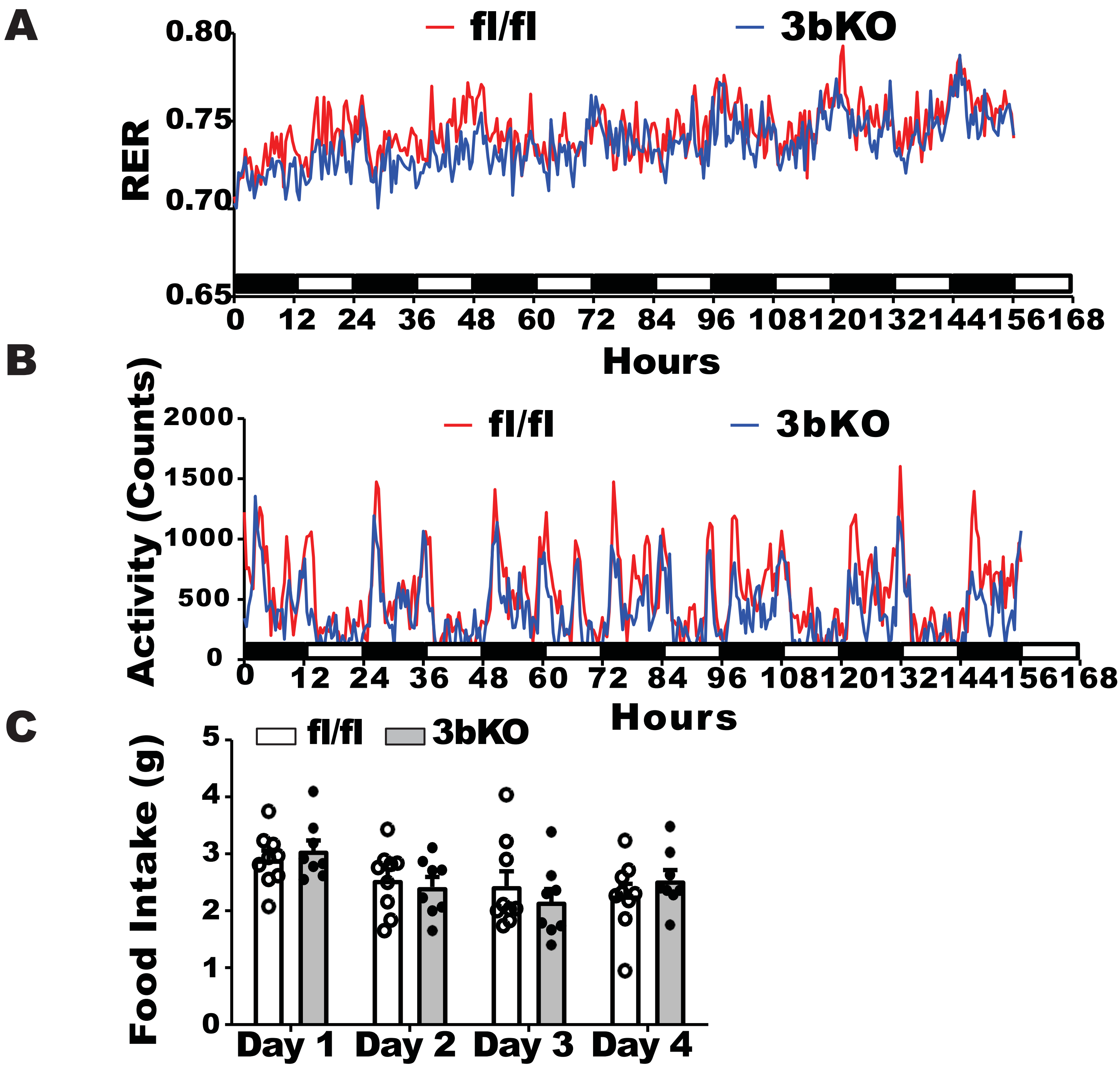

Supplemental Figure S5

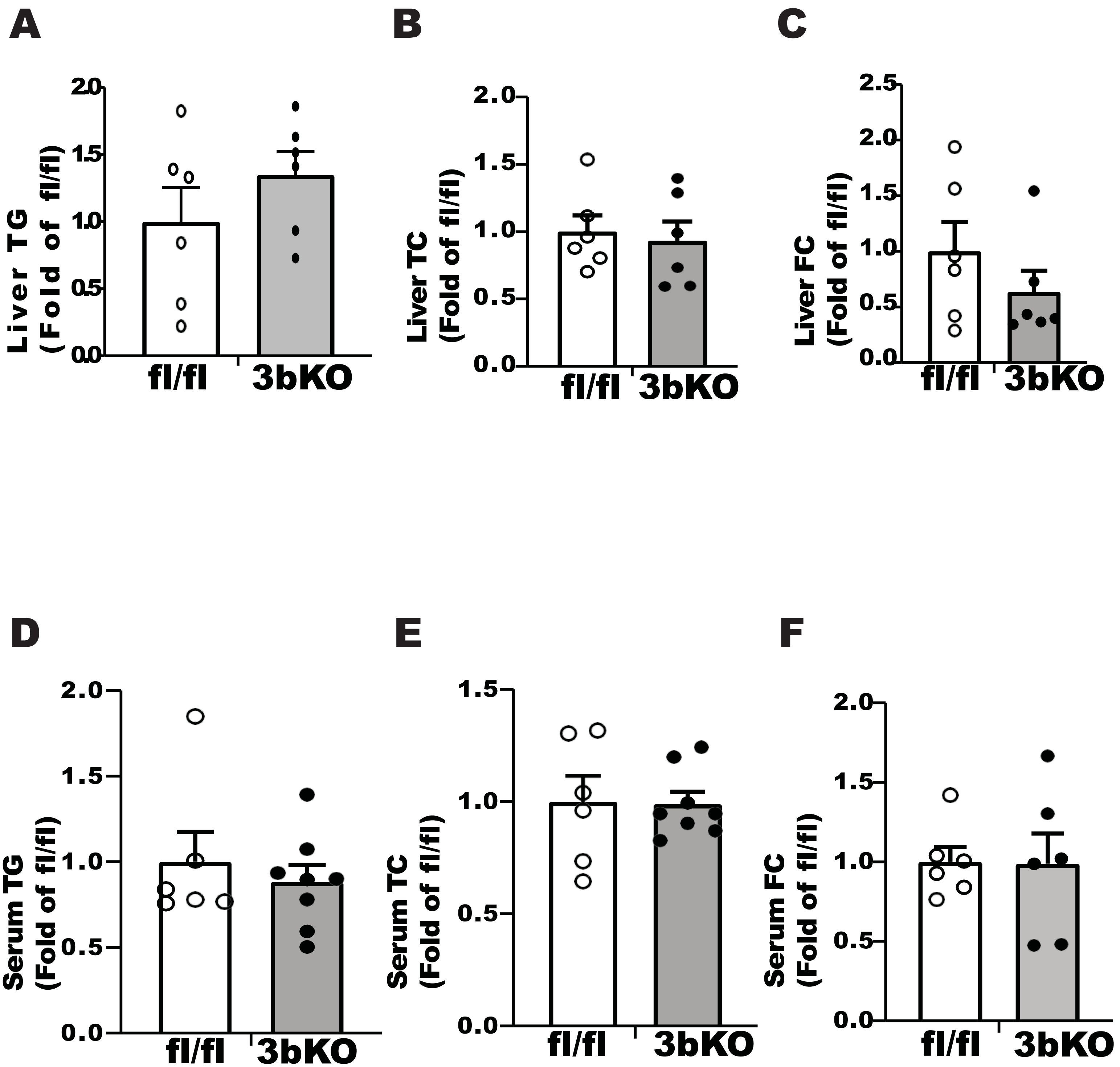

Supplemental Figure S6

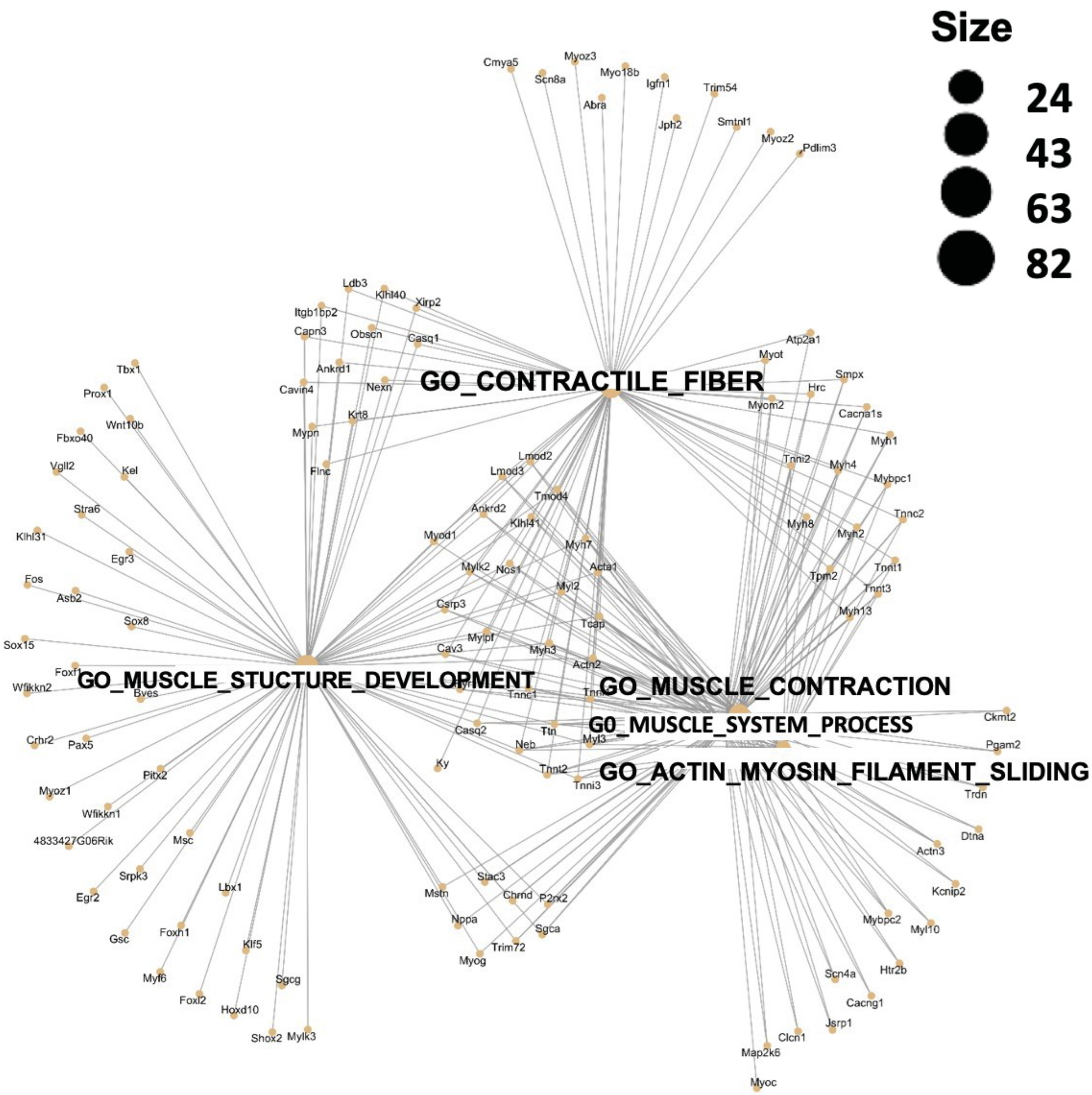

Supplemental Figure S7

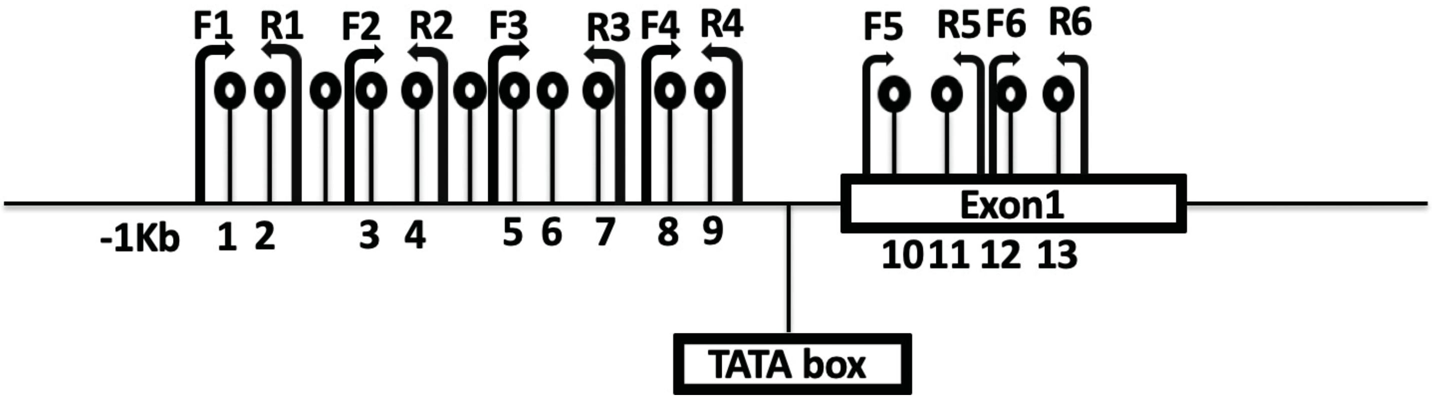

Supplemental Figure S8

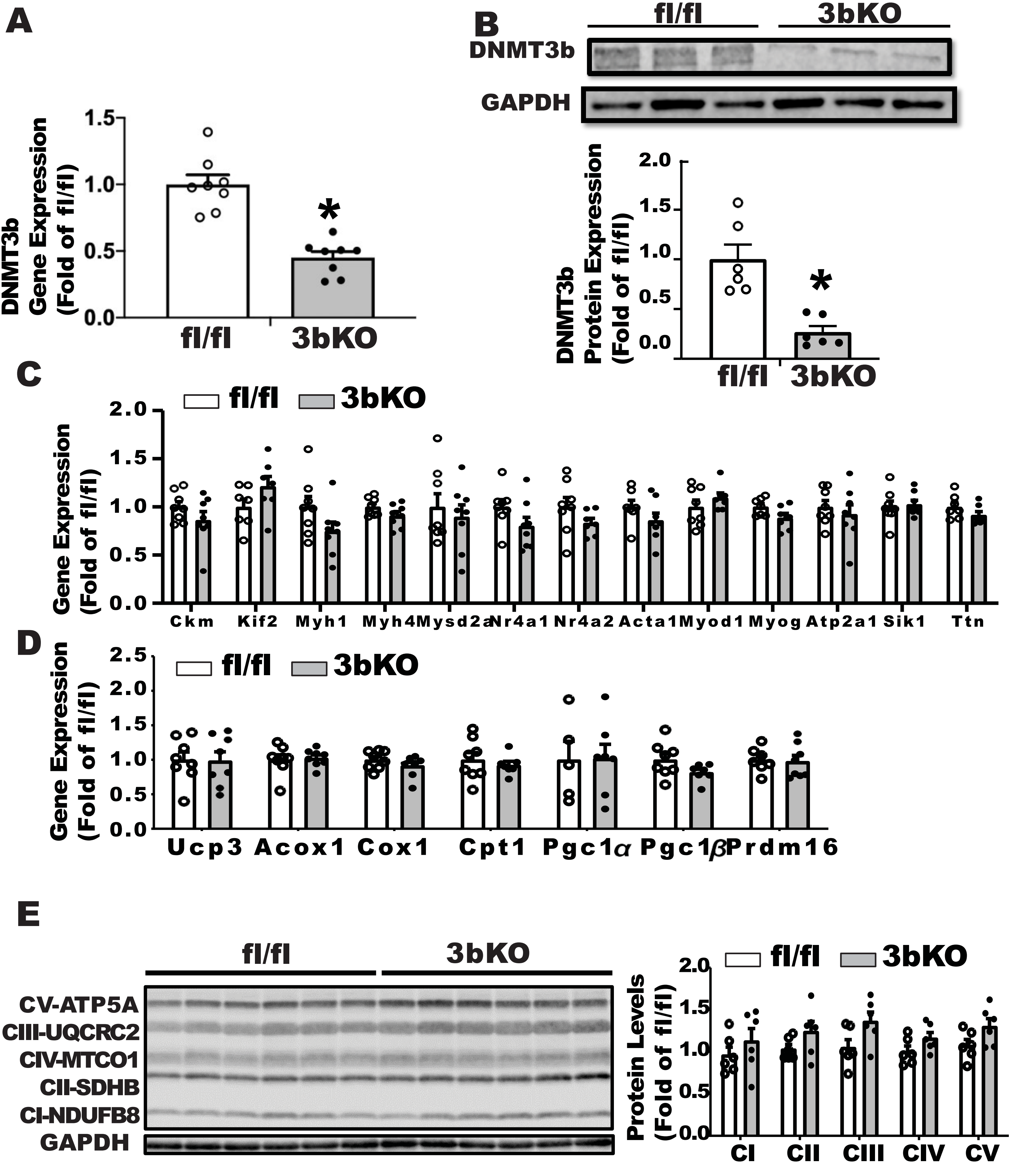

Supplemental Figure S9

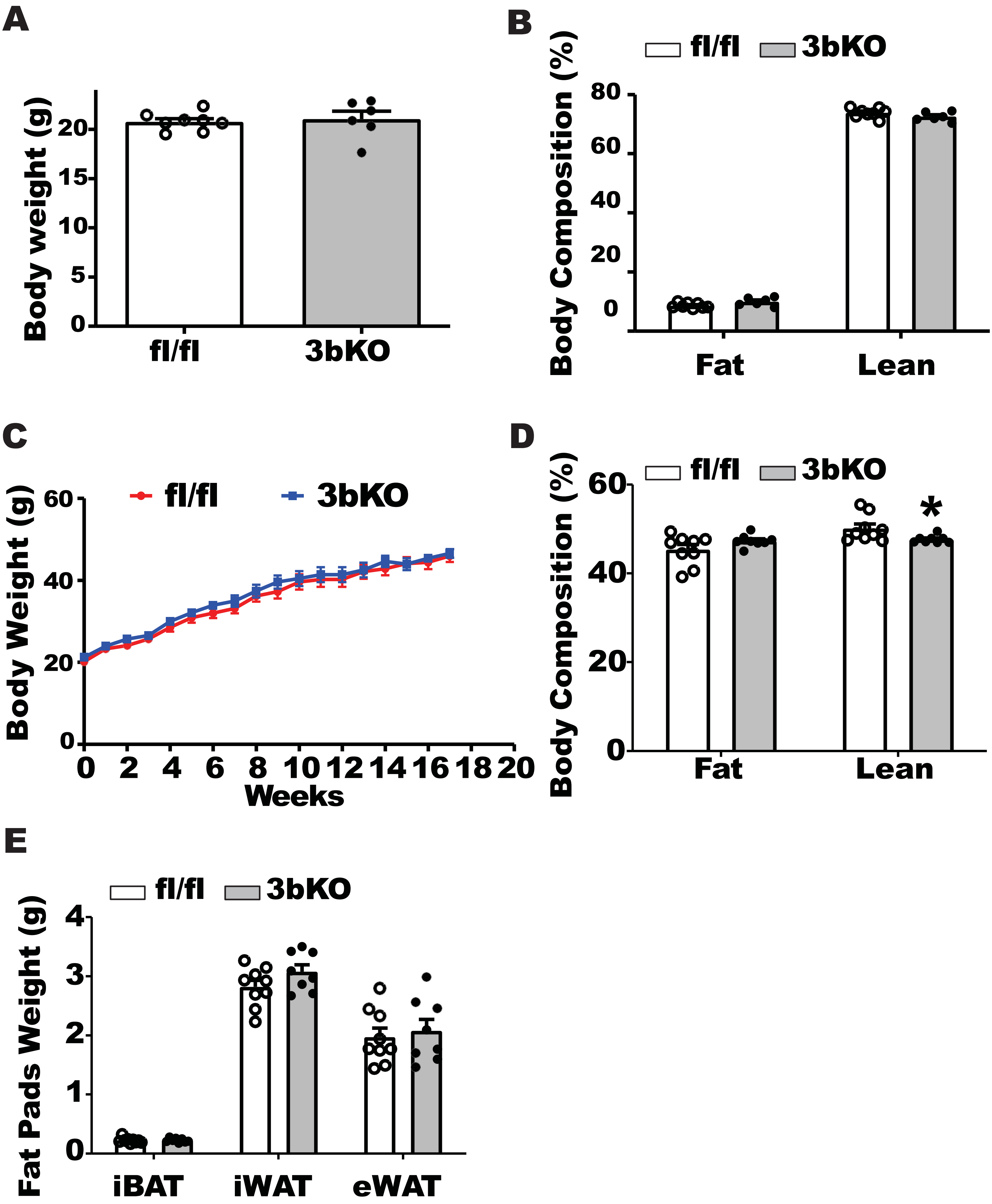

Supplemental Figure S10

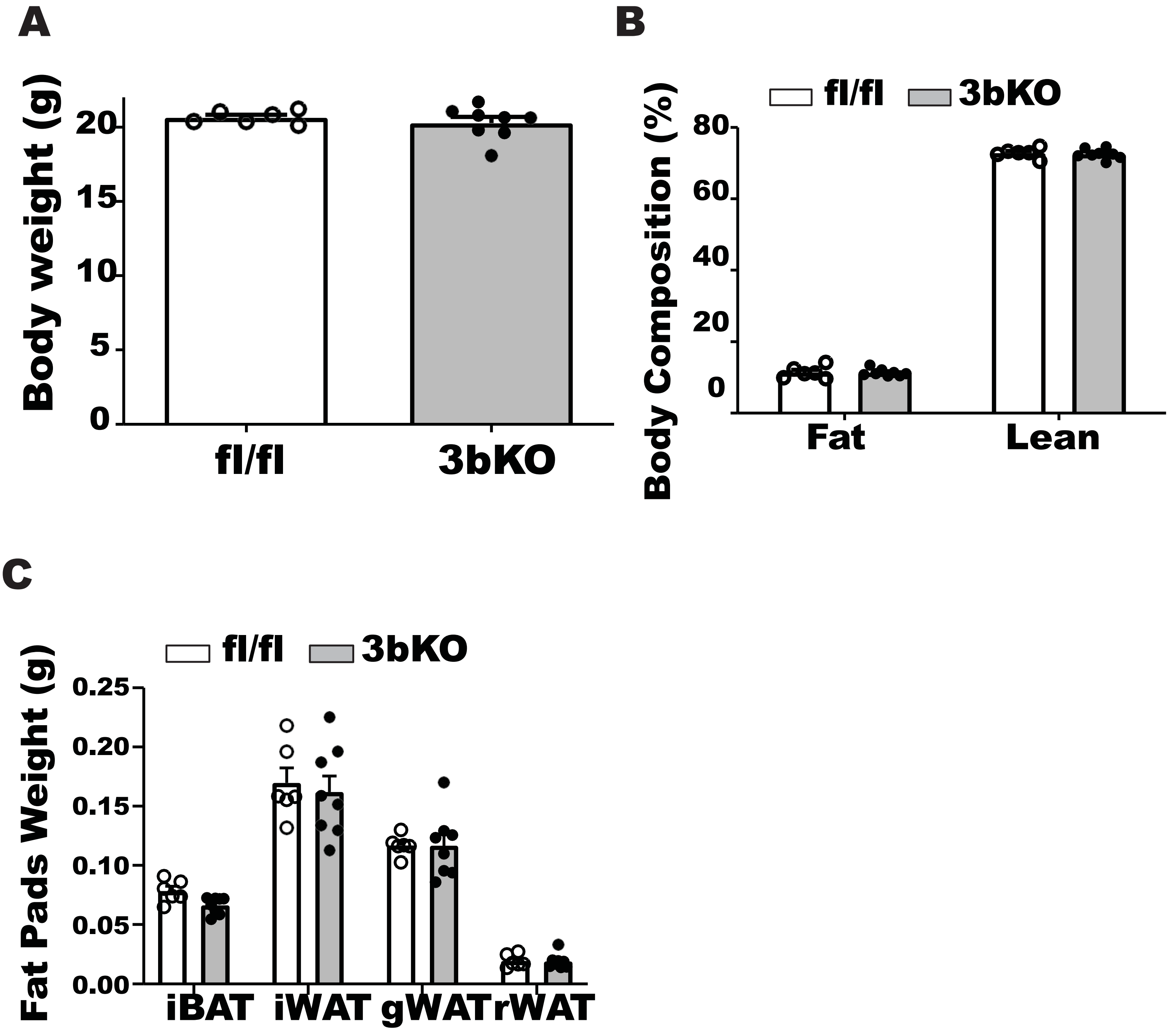

Supplemental Figure S11

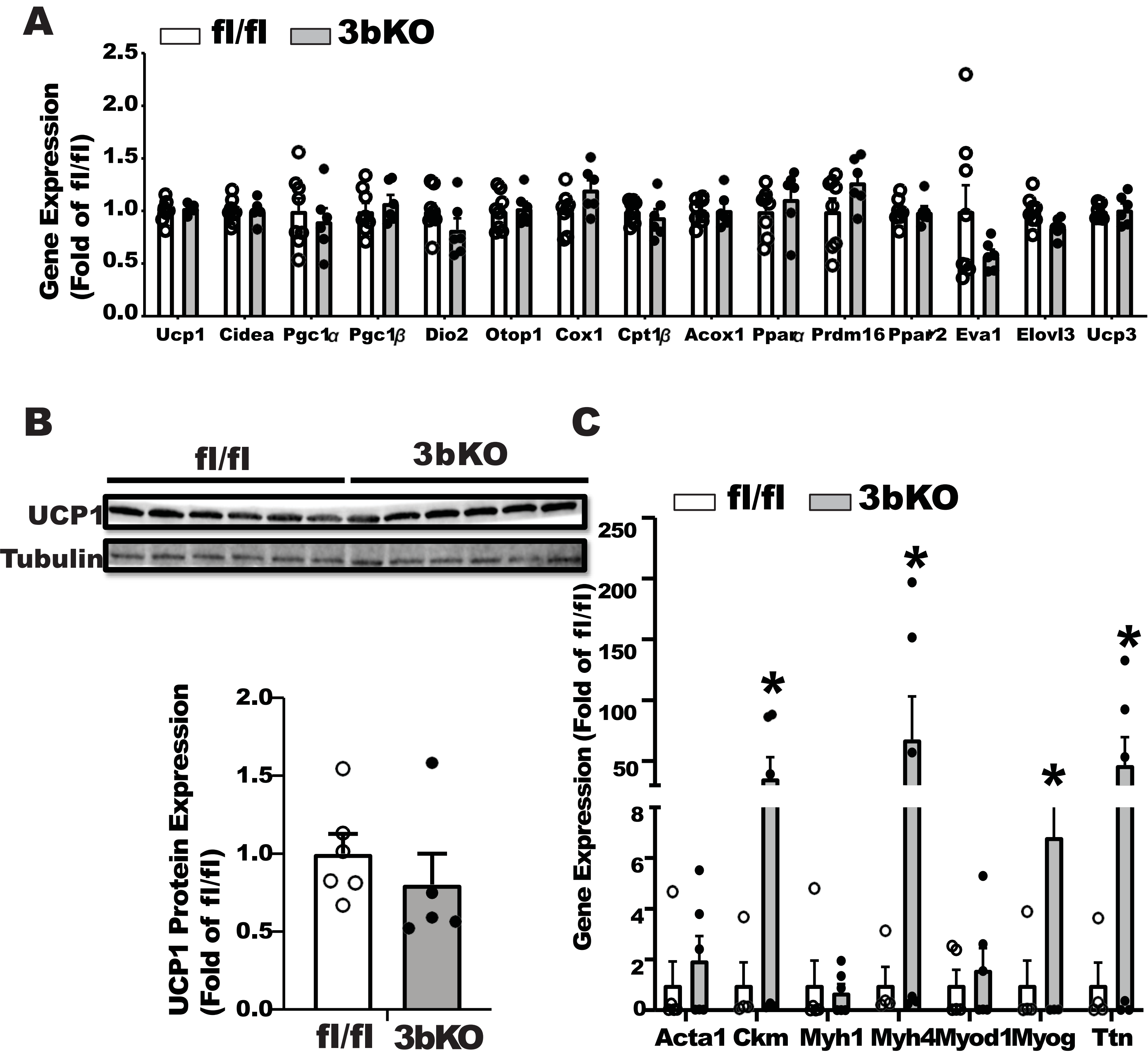

Supplemental Table S1: TaqMan primer/probe pairs purchased from Applied Biosystems

Gene Symbol	Company	Catalog #
Ckm	ABI	Mm01321487_m1
Klf2	ABI	Mm00500486_g1
Myh1	ABI	Mm01332489_m1
Myh4	ABI	Mm01332541_m1
Mysd2a	ABI	Mm01192208_m1
Nr4a1	ABI	Mm01300401_m1
Nr4a2	ABI	Mm00443060_m1
Acta1	ABI	Mm00808218_m1
Myod1	ABI	Mm00440387_m1
Myog	ABI	Mm00446194_m1
Atp2a1	ABI	Mm01275320_m1
Sik1	ABI	Mm00440317_m1
Ttn	ABI	Mm00621005_m1
Dnmt3b	ABI	Mm01240111_g1
Acox1	ABI	Mm01246834_m1
Cidea	ABI	Mm00432554_m1
Cox1	ABI	Mm04225234_m1
Dio2	ABI	Mm00515644_m1
Pgc1a	ABI	Mm01208835_m1
Pgc1b	ABI	Mm00504720_m1
Prdm16	ABI	Mm00712556_m1
Cpt1b	ABI	Mm00487191_g1
Otop1	ABI	Mm00554705_m1
Pparγ	ABI	Mm00440945_m1
Pparγ2	ABI	Mm00440940_m1
Eva1	ABI	Mm00468397_m1
Elovl3	ABI	Mm01194165_g1

Supplemental Table S2. Amplification and Sequencing primers for *Mef2c* pyrosequencing

Primers	Sequences
Amplification primer 1: Forward	5'- TGATGGAGAGGTTGGGATTAA -3'
Amplification primer 1: Reverse	5'- TCAACAAACCTCATTTCCCTACA-3'
Amplification primer 2: Forward	5'- ATGATTGTTAAAGTGGAGTTTTATAAGA -3'
Amplification primer 2: Reverse	5'- AAACCCACAATACTACTATACCA -3'
Amplification primer 3: Forward	5'- GAGTTGGATTGTTAAATTTGTGTTAGAT -3'
Amplification primer 3: Reverse	5'- CATACTCCCAAATTAAAAAACTTATAACTC -3'
Amplification primer 4: Forward	5'- TGGGAGTATGATTAATTTTTTTTATGTGAT -3'
Amplification primer 4: Reverse	5'- CCCAAACCTCTACACTATTAATTCCA -3'
Amplification primer 5: Forward	5'- GTATTGATAAAGGTTTGGTTGTTAATGA -3'
Amplification primer 5: Reverse	5'- AACTTTAAAAAAAAACCCCCCAAT-3'
Amplification primer 6: Forward	5'- GTGTATTTTGGTTTGTTAGATATTTGTGTA -3'
Amplification primer 6: Reverse	5'- AACTTTAAAAAAAAACCCCCCAAT -3'
Sequencing primer 1	5'- ATGAGGAAATTTAAGGGT -3'
Sequencing primer 2	5'- GTGGAGTTTTATAAGATTTTGT -3'
Sequencing primer 3	5'- AATTAGGGTTATATATTAAGGG -3'
Sequencing primer 4	5'- TTTGGATTGAAAAAAGTAAA -3'
Sequencing primer 5	5'- GGTGTATTTTGGTTTGTTAG -3'
Sequencing primer 6	5'- GTTTGACGATTAAGGGGG -3'
